# Supplementary material for: Content-rich biological network constructed by mining PubMed abstracts
Source: BMC Bioinformatics. 2004 Oct 8;5:147. doi: 10.1186/1471-2105-5-147 (PMC528731; doi:10.1186/1471-2105-5-147)
Supplement: Additional File 5 — The original Chilibot query results of the term "long-term potentiation (LTP)" and 22 other terms, limiting the latest references analyzed to the years 1990, 1995, 2000, and 2004. [file 1471-2105-5-147-S5.bz2 › chilibotAdditionalFile5/ltp2004/html/ACTIN_ACTININ.html]

 


 **ACTIN** and **ACTININ** 
  
Found 1125 abstracts in PubMed,  **30 abstracts were retrieved and analyzed**.  


---

 Search Google  |
 PDF files only 
|  EDU domain only 

---

**Interactive relationship** (e.g. stimulation, inhibition, etc)

- Structure of  **actin**  cross linked with alpha  **actinin**  a network of bundles.  Ref: 14611558 Phys Rev Lett, 2003
- Phosphoinositides bind to and regulate the interaction of alpha  **actinin**  with  **actin**  filaments and integrin receptors.  Ref: 14670080 Biochem JBiochem J, 2003
- Myotilin is a Z disc protein that binds alpha  **actinin** , gamma filamin and F  **actin** .  Ref: 12899871 Neuromuscul Disord, 2003
- Immunogold labeling for  **actin**  binding proteins demonstrated that alpha  **actinin**  and synaptopodin were localized in the  **actin**  bundle.  Ref: 14623927 J Histochem Cytochem, 2003
- alpha  **Actinin**  is a cell adhesion and cytoskeletal protein that bundles  **actin**  microfilaments and directly links these filaments to integrin adhesion receptors.  Ref: 14670080 Biochem JBiochem J, 2003
- In the latter role, it binds zyxin and alpha  **actinin** , both of which are involved in  **actin**  organization.  Ref: 14567970 Mol Genet MetabMol Genet Metab, 2003
- Using immunoprecipitation, mass spectroscopy, and confocal microscopy, we demonstrated in normoxia... an interaction between iNOS and alpha  **actinin**  4, an adapter protein that anchors enzymes to the  **actin**  cytoskeleton.  Ref: 12960352 J Immunol, 2003
- CRP2 was associated with the  **actin**  cytoskeleton and decorated  **actin**  stress fibres in a continuous fashion, unlike the periodic labelling pattern observed for alpha  **actinin**  and zyxin, which also accumulate in focal adhesions.  Ref: 14741346 FEBS Lett, 2004
- Filamin cross links F  **actin**  into networks that are more resilient, stiffer, more solid like, and less dynamic than alpha  **actinin**  and fascin.  Ref: 14594947 J Biol Chem, 2004
- the association with the  **actin**  cytoskeleton is independent of alpha  **actinin**  or zyxin localisation in the cell, thus questioning the role of CRP2 as a regulator of alpha  **actinin**  function in vivo.  Ref: 14741346 FEBS Lett, 2004
- A CCM motor domain without light chain binding site moved  **actin**  filaments at a velocity of 8.8 microm s at 30 degrees C and a CCM motor domain with an artificial lever arm consisting of two alpha  **actinin**  repeats moved  **actin**  filaments at 16.2 microm s.  Ref: 14651964 Biochem Biophys Res Commun, 2003
- Using quantitative rheological methods that avoid the pitfalls of previous studies, we systematically tested the complex mechanical response of reconstituted  **actin**  filament networks containing a wide range of filamin concentrations and compared the mechanical function of filamin with that of the cross linking bundling proteins alpha  **actinin**  and fascin.  Ref: 14594947 J Biol Chem, 2004
- Three dimensional laser scanning confocal microscopy has revealed that filamentous  **actin** , when complexed with the cross linking protein alpha  **actinin** , will spontaneously assemble on a micron scale into a structure comprised of a relatively rigid, frequently branching, 3D network of bundles with characteristic mesh size of the order of the persistence length of F  **actin** .  Ref: 14611558 Phys Rev Lett, 2003
- The PDZ LIM protein RIL modulates  **actin**  stress fiber turnover and enhances the association of alpha  **actinin**  with F  **actin** .  Ref: 14729062 Exp Cell Res, 2004
- The organization of the cytoskeletal proteins, vinculin, talin, and alpha  **actinin** , which mediate linkages between  **actin**  filaments and transmembrane integrins, is also regulated by contractile stimulation in airway smooth muscle.  Ref: 14516723 Respir Physiol Neurobiol, 2003

**Parallel relationship** (e.g. studied together, co-existance, homology, etc.)

- Examination of cell adhesion related genes revealed upregulation of c Jun, alpha  **actinin** ,  **actin** , myosin light chain, p120cas catenin Catns, alpha integrin, integrin beta5, fibronectin, IQGAP1, and mCalpain.  Ref: 14580254 Breast Cancer Res, 2003
- The  **actin**  cross linkers alpha  **actinin**  and ABP 120 do not appear to play as major a role as myosin II in providing this cortical integrity.  Ref: 12890752 J Cell Sci, 2003
- Vinculin is a conserved component and an essential regulator of both cell cell cadherin mediated and cell matrix integrin talin mediated focal adhesions junctions, and it anchors these adhesion complexes to the  **actin**  cytoskeleton by binding to talin in integrin complexes or to alpha  **actinin**  in cadherin junctions.  Ref: 14702644 Nature, 2004
- The closely related CLP 36 is predominantly expressed in nonmuscle cells, where it localizes to  **actin**  stress fibers also in association with alpha  **actinin** .  Ref: 14729062 Exp Cell Res, 2004
- Confocal microscopy of hearts of E8 8.5 Tmod1 null embryos revealed structures resembling nascent myofibrils with continuous F  **actin**  staining and periodic dots of alpha  **actinin** , indicating that I Z I complexes assembled in the absence of Tmod1.  Ref: 14657235 J Cell Biol, 2003
- Thus,  **actin** , alpha  **actinin** , MAPK and vinculin may be involved in the regulation of TGC migration.  Ref: 14727175 Cell Tissue ResCell Tissue Res, 2003
- The  **actin**  and alpha  **actinin**  immunoreaction occurred as a band of electron dense particles beneath the cytoplasmic membrane.  Ref: 14727175 Cell Tissue ResCell Tissue Res, 2003
- We have recently demonstrated that the skeletal muscle  **actin**  binding protein alpha  **actinin**  3 is absent in 18% of healthy white individuals because of homozygosity for a common stop codon polymorphism in the ACTN3 gene, R577X. alpha  **Actinin**  3 is specifically expressed in fast twitch myofibers responsible for generating force at high velocity.  Ref: 12879365 Am J Hum Genet, 2003
- **Actin**  and alpha  **actinin**  were co localized apically in mononuclear trophoblast cells, along the cytoplasmic membrane of TGC and apically in maternal crypt cells.  Ref: 14727175 Cell Tissue ResCell Tissue Res, 2003
- Double labelled immune fluorescence was used to detect adherent H. pylori and the distribution of F  **actin** , alpha  **actinin**  and Arp3.  Ref: 12972579 J Med Microbiol, 2003
- Changes in expression of slit pore constituents podocin, CD2AP, nephrin and ZO 1, cytoskeleton associated proteins  **actin** , alpha  **actinin** , ezrin and synaptopodin, the GDH podocyte adhesion molecules alpha 3 integrin, and heparan sulfate were studied by immunofluorescence.  Ref: 14605274 Nephrol Dial Transplant, 2003
- In contrast to the  **actin**  binding proteins SM22alpha and alpha  **actinin** , GFP CRP2 did not translocate into the podosomes induced by this treatment, but remained preferentially bound to the stress fibres, suggesting an  **actin**  filament stabilising role for CRP2.  Ref: 14741346 FEBS Lett, 2004
- Z bands in different muscles have a modular structure formed by layers of alpha  **actinin**  molecules cross linking  **actin**  filaments.  Ref: 12946354 J Mol Biol, 2003
- Over the subsequent 10 min prior to anaphase B, this broad band of proteins condenses into a contractile ring including  **actin** , tropomyosin Cdc8p, and alpha  **actinin**  Ain1p.  Ref: 14602073 Dev Cell, 2003
- To evaluate sarcolemmal integrity, we used an Evans blue dye EBD assay, and to assess structural changes, we used immunofluorescent labeling with antibodies against contractile myosin,  **actin** , cytoskeletal alpha  **actinin** , desmin, dystrophin, beta spectrin, integral membrane alpha and beta dystroglycan, sarcoglycan, and extracellular laminin, fibronectin proteins.  Ref: 14522817 Am J Physiol Cell Physiol, 2004
- Alpha  **actinin** , an  **actin**  binding protein localized in glomerular podocytes, is the major target of nephritogenic anti DNA antibodies.  Ref: 12849011 Autoimmun Rev, 2002
- These effects may be mediated through the association of RIL with alpha  **actinin** , as RIL was found to associate with alpha  **actinin**  via its PDZ domain, and RIL enhanced the ability of alpha  **actinin**  to cosediment with  **actin**  filaments.  Ref: 14729062 Exp Cell Res, 2004
- Downregulation of several key  **actin**  binding proteins, such as alpha  **actinin** , vinculin, gelsolin and tropomyosins TMs, is considered to contribute to the disorganized cytoskeleton present in many neoplastic cells.  Ref: 14722123 J Biol ChemJ Biol Chem, 2004
- We show that newly formed complexes are located posterior to an  **actin** , VASP and alpha  **actinin**  rich region in the lammelipodium.  Ref: 14576354 J Cell Sci, 2003
- We tested the proposed model by expressing deletion mutants of N RAPi.e. constructs containing two of the three regions of N RAP in chick cardiomyocytes and observing the effects on alpha  **actinin**  and  **actin**  organization into mature sarcomeres.  Ref: 14657273 J Cell Sci, 2004
- In addition, a protease sensitivity assay was developed to examine the flexibility of the linker region between the  **actin**  binding domain and the spectrin repeats of alpha  **actinin** .  Ref: 14670080 Biochem JBiochem J, 2004
- Based on biochemical data, immunofluorescence analysis of cultured embryonic chick cardiomyocytes and the targeting and phenotypic effects of these individual GFP tagged regions of N RAP, we proposed a novel model for the initiation of myofibril assembly in which N RAP organizes alpha  **actinin**  and  **actin**  into the premyofibril I Z I complexes.  Ref: 14657273 J Cell Sci, 2004
- Immunofluorescence labeling for  **actin**  binding proteins in immature podocyte showed that alpha  **actinin**  was localized at the level of the junctional complex.  Ref: 14623927 J Histochem Cytochem, 2003
- We monitored the expression of cytoskeletal and cell adhesion proteins  **actin** , myosin, desmin, alpha  **actinin** , troponin, titin, vimentin and vinculin using immunofluorescence microscopy and video enhanced, background subtracted, differential interference contrast of 24 to 48 h zebrafish embryos.  Ref: 12886467 Braz J Med Biol Res, 2003
- In cow placentomes,  **actin** , its associated proteins alpha  **actinin** , vinculin and a key protein kinase of the signal transduction cascade phosphorylated mitogen activated protein kinase, pMAPK were localized by immunogold silver enhancement and immunoperoxidase staining at the light and transmission electron microscopical levels.  Ref: 14727175 Cell Tissue ResCell Tissue Res, 2003
